# The Potential of Mesenchymal Stromal Cell as Therapy in Neonatal Diseases

**DOI:** 10.3389/fped.2020.591693

**Published:** 2020-11-04

**Authors:** Ling Ling Liau, Maimonah Eissa Al-Masawa, Benson Koh, Qi Hao Looi, Jhi Biau Foo, Sau Har Lee, Fook Choe Cheah, Jia Xian Law

**Affiliations:** ^1^Department of Physiology, Faculty of Medicine, Universiti Kebangsaan Malaysia Medical Centre, Kuala Lumpur, Malaysia; ^2^Centre for Tissue Engineering and Regenerative Medicine, Faculty of Medicine, Universiti Kebangsaan Malaysia Medical Centre, Kuala Lumpur, Malaysia; ^3^Future Cytohealth Sdn Bhd, Bandar Seri Petaling, Kuala Lumpur, Malaysia; ^4^School of Pharmacy, Faculty of Health and Medical Sciences, Taylor's University, Subang Jaya, Malaysia; ^5^School of Biosciences, Faculty of Health and Medical Sciences, Taylor's University, Subang Jaya, Malaysia; ^6^Department of Paediatrics, Faculty of Medicine, Universiti Kebangsaan Malaysia Medical Centre, Kuala Lumpur, Malaysia

**Keywords:** cell therapy, clinical trial, neonatal diseases, extracellular vesicles, mesenchymal stromal cells

## Abstract

Mesenchymal stromal cells (MSCs) can be derived from various tissue sources, such as the bone marrow (BMSCs), adipose tissue (ADSCs), umbilical cord (UC-MSCs) and umbilical cord blood (UCB-MSCs). Clinical trials have been conducted to investigate the potential of MSCs in ameliorating neonatal diseases, including bronchopulmonary dysplasia (BPD), intraventricular hemorrhage (IVH) and necrotizing enterocolitis (NEC). In preclinical studies, MSC therapy has been tested for the treatment of various neonatal diseases affecting the heart, eye, gut, and brain as well as sepsis. Up to date, the number of clinical trials using MSCs to treat neonatal diseases is still limited. The data reported thus far positioned MSC therapy as safe with positive outcomes. However, most of these trials are still preliminary and generally smaller in scale. Larger trials with more appropriate controls and a longer follow-up period need to be conducted to prove the safety and efficacy of the therapy more conclusively. This review discusses the current application of MSCs in treating neonatal diseases, its mechanism of action and future direction of this novel therapy, including the potential of using MSC-derived extracellular vesicles instead of the cells to treat various clinical conditions in the newborn.

## Introduction

Mesenchymal stromal cells (MSCs) possess several unique properties which render them an ideal candidate for cell-based therapy in various neonatal diseases. MSCs are multipotent and can migrate to the damaged tissues or organs in response to the inflammatory mediators. At the injured sites, the cells can replicate and differentiate to regenerate the loss or damaged tissues. Besides, MSCs also secrete paracrine factors such as growth factors, cytokines and chemokines which diffuse to local tissue environment and interact with the surrounding cells. These paracrine factors can remedy tissue injury and inflammation as well as mediate tissue repair and regeneration by exerting its anti-inflammatory, anti-apoptotic and pro-mitotic effects (1–3).

MSCs are classified into various groups according to the cell source, such as bone marrow-derived MSCs (BMSCs), adipose-derived MSCs (ADSCs), umbilical cord-derived MSCs (UC-MSCs), and umbilical cord blood-derived MSCs (UCB-MSCs) (4). BMSCs were favored by some researchers since the cells are proven to be safe, easily accessible and can be extracted from the patient's bone marrow (autologous) thus minimizing the risk of immune rejection (5, 6). On the other hand, UC-MSCs and UCB-MSCs appear as attractive sources of allogeneic MSCs due to their availability and immune evasive nature (7–9). Generally, the procedure of BMSC preparation is more time consuming and costlier compared to UC-MSCs and UCB-MSCs, thus the use of UC-MSCs and UCB-MSCs may be preferred.

Regardless of the tissue sources, MSCs have low immunogenicity and can suppress the activation of immune cells (10). Therefore, both autologous and allogeneic MSCs can be used clinically as the cells have a very low risk of being rejected by the host immune system. A significant advantage of allogeneic MSCs is that the cells can be developed into an off-the-shelf product that is convenient to be used clinically. Table 1 highlights the advantages and disadvantages of different MSCs.

**Table 1:** Comparison between BMSCs, ADSCs, UC-MSCs and UCB-MSCs for stem cell therapy.

**Characteristic**	**BMSCs**	**ADSCs**	**UC-MSCs UCB-MSCs**
Ethical conundrum	No	No	No
Tissue collection	Invasive	Invasive	None invasive (unwanted tissue after delivery)
Tissue processing and cell culture	Easy	Easy	Easy
Effect of donor age on cells	Quantity and quality decline with age	Quantity and quality decline with age	Unaffected
Cell proliferative potential	Lower	Lower	Higher
Expression of embryonic markers	Lower	Lower	Higher
Anti-inflammatory property	Good	Good	Good
Allogeneic cell rejection	No	No	No
Risk of tumor formation	Very low	Very low	Very low

*BMSCs, Bone marrow-derived mesenchymal stromal cells; ADSCs, adipose tissue-derived mesenchymal stromal cells; UC-MSCs, umbilical cord-derived mesenchymal stromal cells; UCB-MSCs, Umbilical cord blood-derived mesenchymal stromal cells*.

**Figure 1:**
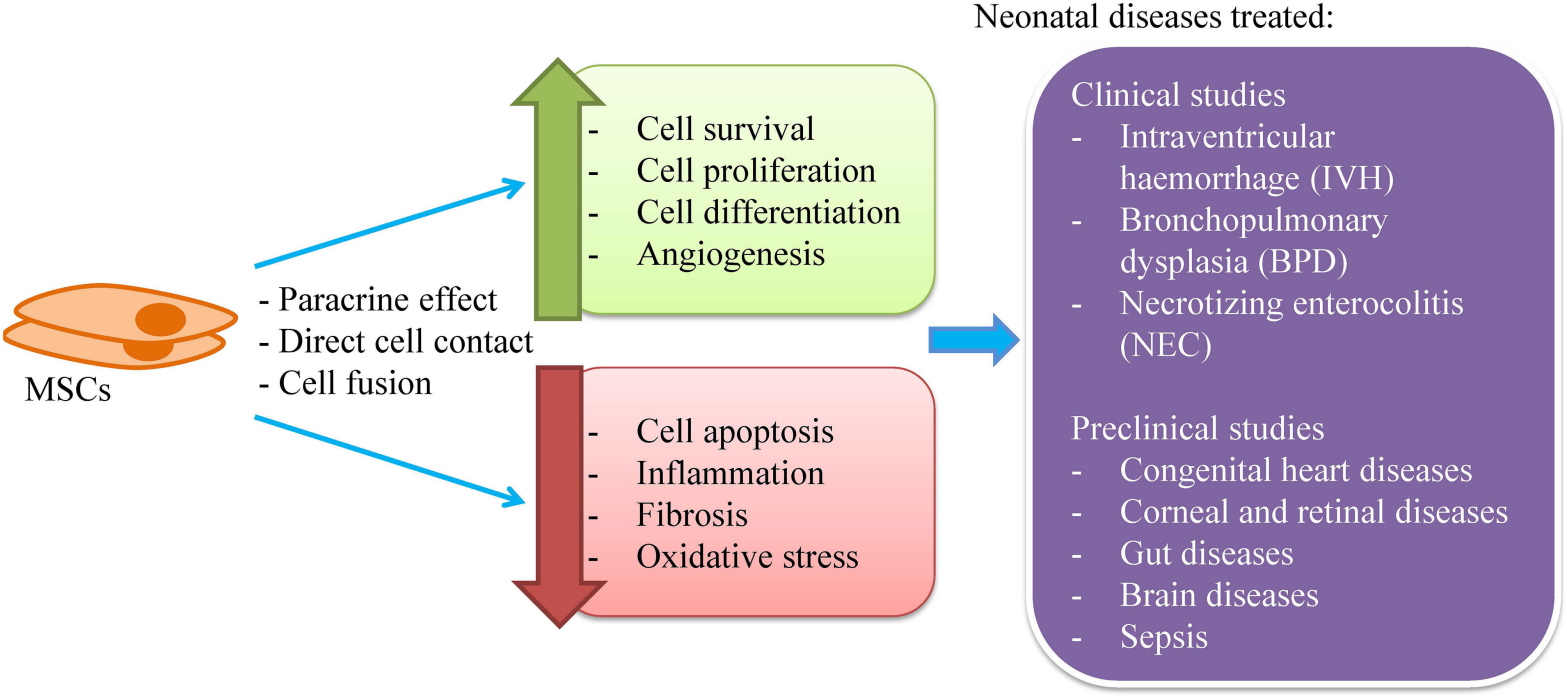
MSCs for the treatment of neonatal diseases. MSCs are known to exert its therapeutic effect through paracrine secretion, direct cell contact and cell fusion to enhance cell survival, proliferation and differentiation, and angiogenesis as well as to reduce cell apoptosis, inflammation, fibrosis, and oxidative stress. Clinically, MSCs have been used to treat intraventricular hemorrhage, bronchopulmonary dysplasia, and necrotizing enterocolitis. Besides, MSCs have been tested for the treatment of congenital heart diseases, eye, gut, and brain diseases as well as sepsis.

MSC therapy can be instituted during the first month of life and is reportedly quite safe. MSC therapy has been introduced to treat neonatal diseases such as severe intraventricular hemorrhage (IVH), bronchopulmonary dysplasia (BPD), and necrotizing enterocolitis (NEC) (11–13) (Figure 1). MSCs are more widely used clinically to treat neonatal diseases as the cells do not have ethical concerns and have a very low risk of tumor formation compared to the embryonic stem cells (ESCs) and induced pluripotent stem cells (iPSCs). In preclinical studies, MSCs have been experimented to treat congenital heart, eye, gut and brain diseases as well as sepsis.

In this review, literature search was conducted using the keywords (1) neonate and (2) clinical and (3) mesenchymal stem cells on Pubmed and Medline databases and all the relevant articles were included in the review.

## MSCs for the Treatment of Neonatal Lung Diseases

### Bronchopulmonary Dysplasia (BPD)

BPD is a chronic lung disease affecting mostly preterm neonates and is characterized by disrupted alveolar growth and pulmonary vascularization. Recent years have witnessed an increased incidence of BPD due to the spike in the population of preterm survivors as a result of the improvement in neonatal care. Pulmonary hypertension is common in severe cases of BPD. Affected infants may require oxygen support and commonly develop respiratory problems that need hospitalization (14, 15).

Currently, clinical trials evaluating MSC therapeutic and preventive effects on BPD are still limited (Table 2). The majority of the studies are small in scale, non-randomized and lack proper comparison groups. To the best of our knowledge, the first clinical study was reported by Chang et al. in 2014 which followed-up the patients up to 2 years (11, 16). The study examined the safety and feasibility of single intratracheal transplantation of 1 × 10^7^ and 2 × 10^7^ allogeneic UCB-MSCs/kg in 9 preterm infants with a high risk of developing BPD at the age of 7–14 days. Results showed that UCB-MSC therapy helped to reduce BPD severity and inflammatory markers as well as to minimize the risk of neurodevelopmental morbidities. Powell et al. treated 12 preterm infants with BPD using the same protocol as Chang et al. and found that disease severity improved after the stem cell treatment (19). Alvarez-Fuente et al. reported suppression of pro-inflammatory as well as improvement in pulmonary hypertension and diminished levels of surfactant protein D (SP-D) expression, a biomarker for lung injury, in 2 preterm infants given multiple dosages of allogeneic BMSCs intravenously (17). Nonetheless, both patients received stem cell therapy at the very advanced stages of BPD and passed away 6 weeks after the initiation of MSC therapy. Lin et al. reported improvement in respiratory functions after intratracheal administration of maternal BMSC (6.25 × 10^6^ cells/kg) in a 10-month old preterm infant with BPD that developed acute respiratory distress syndrome (ARDS) (18). Results from these clinical studies suggested that the beneficial effects of MSCs might be attributed to paracrine effects that stimulate alveolarization and vascularization rather than cell engraftment and proliferation as traces of donor cells in the recipients were absent.

**Table 2:** Clinical trials used MSCs to treat neonatal diseases.

**References/year/study location**	**ClinicalTrials.gov identifier**	**Study design**	**Disease/pathology**	**Age group**	**No. of patient**		**Cell source**	**Route of administration**	**Dose**	**Frequency of cell administration**	**Follow up period**	**Safety outcome**	**Key efficacy outcome**
					**C**	**T**							
Chang et al. (11), 2014, South Korea	NCT01297205	Phase 1 dose-escalation clinical trial	BPD	5 to 14-day old (23–29 gestational weeks)	0	9	Allogeneic UCB-MSCs (Pneumostem®)	Intratracheal	Group A: 3 patients received 1 × 10^7^ cells/kg Group B: 6 patients received 2 × 10^7^ cells/kg	Single dose	84 days	- No MSC related AE - No dose-limiting toxicities - No tumor formation	- BPD severity reduced - Retinopathy of prematurity demanding surgery occurred less in the MSC-treated group compared to the historical corresponding control group - Significant declined in IL-6, IL-8, MMP-9, TNF-a, and TGF-b1 levels in tracheal aspirates at day 7
Ahn al. (16), 2014, South Korea	NCT01632475	Phase I trial	BPD	5 to 14-day old (23–29 gestational weeks)	0	9	Allogeneic UCB-MSCs (Pneumostem®)	Intratracheal	Group A: 3 patients received 1 × 10^7^ cells/kg Group B: 6 patients received 2 × 10^7^ cells/kg	Single dose	24 months	- No MSC-related AE	- Infants treated with MSCs did not require supplemental oxygen upon discharge compared to 22% in the historical corresponding control group - Infants treated with MSCs did not develop asthma and did not need persistent steroid/bronchodilator therapy as long as 24 months of CA - MSC treatment minimized risk of neurodevelopmental morbidities.
Álvarez-Fuente et al. (17), 2018, Spain	–	Phase 1 trial	BPD	Patient1: 5-month old Patient 2: 85-day old	0	2	Allogeneic BMSCs	Intravenous	Patient1: Increasing weekly dose: 1.1 × 10^6^ cells/kg up to 13.9 × 10^6^ cells/kg Patient 2: 5 × 10^6^ cells/kg per week for 3 consecutive weeks	Patient 2 received 3 doses	–	- No MSC-related AE	- Inflammatory and lung injury biomarkers decreased - MSCs failed to reverse late stage of lung fibrosis, thus patients did not survive
Lin et al. (18), 2018, Taiwan	–	Case study	BPD with ARDS	10-month old	0	1	Allogeneic BMSCs	Intratracheal	6.25 × 10^6^ cells/kg	Single dose	-	- No MSC-related AE	- Improvement in respiratory functions - ECMO support was detached at 25 days after transfusion - Improvement in lung fibrosis
Powell and Silvestri (19), 2019, USA	NCT02381366	Phase 1, open-label, dose-escalation trial	BPD	6 to 14-day old	0	12	Allogeneic UCB-MSCs (Pneumostem®)	Intratracheal	Group A: 6 patients received 1 × 10^7^ cells/kg Group B: 6 patients received 2 × 10^7^ cells/kg	Single dose	Up to 20 months CA	- No severe AEs related to MSCs - No dose-limiting toxicities.	- All patients developed BPD - 10/12 patients developed severe BPD showed improvement at day 84 after transfusion - Potential effectiveness could not be established.
Ahn et al. (12), 2018, South Korea	NCT02274428	Phase I dose-escalation clinical trial	IVH (Grade 4)	11.6 ± 0.9-day old	0	9	Allogeneic UCB-MSCs	Intraventricular	Group A: 3 patients received 5 × 10^6^ cells/kg Group B: 6 patients received 1 × 10^7^ cells/kg	Single dose	NA	- No fatality - No anaphylactic reaction - Severe AEs observed were not related to MSC transplantation	–
Akduman et al. (13), 2019, Turkey	–	Case study	NEC (SVT related)	22-day old	0	1	Allogeneic UC-MSCs	Intravenous	1 × 10^7^ cells/transfusion	Single dose	12 months	- No AE	- Intestinal blood supply improved - Saved the remaining of the necrotic intestine after laparotomy - Helped maintain the baby growth and neurodevelopment on par with babies of his age.

*AE, adverse event; ARDS, acute respiratory distress syndrome; BMSCs, bone marrow derived mesenchymal stromal cells; BPD, bronchopulmonary dysplasia; CA, corrected age; C- control; T, treatment; UC-MSCs, umbilical cord derived mesenchymal stromal cells; UCB-MSCs, umbilical cord blood derived mesenchymal stromal cells; IVH, intraventricular hemorrhage; NEC, necrotizing enterocolitis; SVT, supraventricular tachycardia*.

The optimal dosage regimen, including the optimal MSC per dose, number of doses, timing of transplantation, time between doses, and route of administration, is essential in determining MSC efficacy. However, this regimen has not been established yet. The identified clinical studies used MSC dose of 1–20 × 10^6^ cells/kg body weight. Studies that compared high and low doses (1 × 10^7^ vs. 2 × 10^7^ cells/kg) found no difference in outcome (11, 19). Majority of the studies gave a single dose except for Alvarez-Fuente et al. that applied multiple infusions (17). Chang et al. noticed that respiratory function improvement started to decline after 7 days of transplantation (11). This finding supports the notion of the inadequacy of a single-dose approach and the authors suggested that day 7 could be the time for the second dosage of MSCs. Another issue is determining the optimal timing of MSC transplantation. Generally, researchers agreed that early intervention is more beneficial for BPD patients. Most studies administered the cells using the intratracheal route to ensure high cell homing at the targeted tissue while evading systemic side effects (11, 18, 19). Besides, the airway is easily accessible through the endotracheal tube in preterm neonates assisted with mechanical ventilation. Although there are concerns over the risk of delivering a large number of MSCs to the premature lungs, nonetheless, intratracheal administration has been shown to be safe and feasible. Intravenous administration also has been reported to be safe and this route might be preferred in infants that do not require invasive ventilation (17). Preclinical studies showed that intravenously administered MSCs reach the lungs within 24 h and dissipate in 7 days after infusion with no traces in other organs (20).

In spite of the inconsistencies on the degree of effectiveness of MSCs, all reported studies support the apparent safety profile of MSCs regardless of the dose and route of administration. There was no anaphylactic shock, no immune rejection, no mass formation and no dose-limiting toxicities reported. This has led to much excitement about the potential of using MSCs in the prevention and treatment of BPD. There are many more studies that are currently underway (Table 3).

**Table 3:** Clinical trials using MSCs to treat neonatal diseases recorded in ClinicalTrials.gov.

**ClinicalTrials.gov identifier**	**Cell source**	**Route of administration**	**Age eligibility**	**Disease**	**Phase**	**No. of patient**	**Country**
NCT03356821	BMSCs	Intranasal	newborns with gestational age ≥36 weeks	PAIS	I/II	10	Netherlands
NCT04255147	UC-MSCs	Intravenous	up to 21-day old	BPD	I	9	Canada
NCT03683953	MSCs	Intrathecal	newborns with gestational age between 28 and 37 weeks	BPD	I	200	China
NCT03601416	UC-MSCs	Intravenous	up to 1-year old	BPD	II	57	China
NCT03645525	UC-MSCs	Intratracheal	up to 3-week old	BPD	I/II	180	China
NCT03774537	UC-MSCs	Intravenous	up to 14-day old	BPD	I/II	20	China
NCT01207869	UC-MSCs	Intrathecal	up to 6-month old	BPD	I	10	China
NCT03631420	UC-MSCs	–	36 to 48-week old	BPD	I	9	Taiwan
NCT03857841	BMSCs-EVs (UNEX-42)	Intravenous	up to 14-day old	BPD	I	18	United States
NCT02443961	MSCs	–	1-month to 28-week old	BPD	I	10	Spain
NCT04062136	UC-MSCs	Intravenous	1 to 6-month old	BPD	I	10	Vietnam
NCT03873506	UC-MSCs	Intravenous	1-month to 5-year old	BPD	I	30	China
NCT03558334	UC-MSCs	Intravenous	all ages	BPD	I	12	China
NCT03392467	UCB-MSCs (Pneumostem®)	–	up to 13-day old	BPD	II	60	Korea
NCT04003857	UCB-MSCs (Pneumostem®)	Intratracheal	6 to 60-month old	BPD	follow-up of NCT03392467	60	South Korea
NCT01828957	UCB-MSCs (Pneumostem®)	Intratracheal	up to 14-day old	BPD	II	69	South Korea
NCT01897987	UCB-MSCs (Pneumostem®)	Intratracheal	7-month old	BPD	follow-up of NCT01828957	70	South Korea
NCT02023788	UCB-MSCs (Pneumostem®)	Intratracheal	45 to 63-month old	BPD	follow-up of NCT01632475	8	South Korea
NCT03378063	UCB-MSCs	–	1 to 3-month old	BPD	I	100	China
NCT03635450	UC-MSCs	Intravenous	up to 48-hour old	HIE	I	6	United States
NCT02854579	a. hFF-NPCs b. MSCs-derived paracrine factors c. hFF-NPCs + MSCs-derived paracrine factors	Intrathecal	up to 14-day old	HIE	–	120	China
NCT01962233	UC-MSCs	Intravenous	all ages	HIE	I	10	China
NCT03525418	BMSCs	Intramyocardial	up to 1-year old	HLHS	I/II	30	United States
NCT03079401	MPCs	Intramyocardial	up to 5-year old	HLHS	I/II	24	United States
NCT03525418	BMSCs	Intramyocardial	up to 1-year old	HLHS	I/II	30	United States
NCT02855112	ADSCs	Intrathecal	5 to 12-month old	ISMA	I/II	10	Iran
NCT02274428	UCB-MSCs (Pneumostem®)	Intraventricular	23 to 34-week gestational age	IVH	I/II	9	South Korea
NCT02673788	UCB-MSCs (Pneumostem®)	–	6-month to 2-year old	IVH	follow-up of NCT02274428	9	South Korea
NCT02890953	UCB-MSCs (Pneumostem®)	Intraventricular	within 28 postnatal days	IVH	IIa	22	South Korea

*ADSCs, adipose tissue-derived mesenchymal stromal cells; BPD, bronchopulmonary dysplasia; BMSCs, bone marrow-derived mesenchymal stromal cells; CP, cerebral palsy; CM, conditioned medium; EVs, extracellular vesicles; hFF-NPCs, aborted human fetal forebrain-derived neural progenitor cells; HIE, hypoxic-ischemic encephalopathy; HLHS, hypoplastic left heart syndrome; UCB-MSCs, umbilical cord blood-derived mesenchymal stromal cells; UC-MSCs, umbilical cord-derived mesenchymal stromal cells; ISMA, infantile spinal muscular atrophy; IVH, intraventricular hemorrhage; MPC, mesenchymal precursor cells; PAIS, perinatal arterial ischemic stroke; UC-HSCs umbilical cord-derived hematopoietic stem cells; uAVC, unbalanced atrioventricular canal*.

## MSCs For the Treatment of Neonatal Brain Diseases

Neonatal brain diseases, including intraventricular hemorrhage (IVH), neonatal stroke (NS), hypoxic-ischemic encephalopathy (HIE) and periventricular leukomalacia (PVL), are the major contributor to the high mortality and morbidity rates among the neonate (21). Experimental investigations have identified multiple causes for these diseases, including perinatal-induced neuronal cell death due to depleted tissue energy reserve, placental disruption, prolapsed umbilical cord, dysfunctional mitochondria, accumulated free oxygen radical species, persistent inflammatory reactions, defective maturation, and myelination of neuronal cells, as well as the loss of endogenous stem cell pool that is responsible for cell differentiation and tissue repair (22, 23). In the developing brain, pre-oligodendrocytes normally present in high number to form mature oligodendrocytes for the production of myelin sheath. Unfortunately, these pre-oligodendrocytes are more vulnerable than its mature counterpart, hence are more easily lost in the presence of the injurious assaults leading to the development of neonatal brain diseases (22). Despite the advances and progress in neonatal therapeutic medicine, there is still a lack of therapy that is effective to prevent or cure developmental brain injuries in neonates. Therefore, research for a newer, safer and more effective therapeutic approach is necessary to improve the outcome of these conditions. Thus, far, the use of stem cell-based therapy has been well-researched as an intervention to ameliorate complications associated with brain diseases. MSCs have been used clinically to treat IVH, whilst pre-clinical studies have been conducted to unveil the potential of MSCs to attenuate or to repair the brain injury present in various disease models, including NS, HIE, and PVL.

### Intraventricular Hemorrhage (IVH)

IVH is a perinatal brain morbidity that results from the rupture and bleeding of the germinal matrix that is restricted to the ventricular area of the brain. Premature neonates are at a higher risk of developing IVH (24). IVH continues to be one of the leading causes of mortality and lifetime disability in infants. A recent systematic review concluded that prematurity and IVH are major contributing factors to the increased risk for cerebral palsy (CP) in neonates (25).

Clinical research examines MSC therapeutic and preventive effect on IVH is still in its infancy. To the best of our knowledge, the first clinical trial for the evaluation of MSC therapy in IVH patients was revealed by Ahn et al. in South Korea in 2018 (12). It was an uncontrolled phase I dose-escalation study intended to investigate the safety and feasibility of the intraventricular transplantation of allogeneic UCB-MSCs. Nine premature newborns of gestational age 23–34 weeks with severe IVH of grade 3 and 4 were recruited and received a single dose of UCB-MSCs. The average age at transplantation was 11.6 days, around 7 days after IVH diagnosis. UCB-MSCs were injected directly to the lateral ventricle through the anterior fontanelle. Two different UCB-MSC concentrations were tested. The lowest dose used was 5 × 10^6^ cells/kg and the highest dose was 1 × 10^7^ cells/kg given to 3 and 6 patients, respectively. Preliminary findings showed that transplantation was well-tolerated with no immune rejection, anaphylactic shock, dose-related toxicity, death, or any MSC infusion-related severe adverse events. Eight infants developed prematurity related disorders including BPD, sepsis, retinopathy of prematurity, and NEC. Additionally, 5/9 MSC recipients needed shunt placement. The 2-year follow-up study to evaluate the safety of MSC transplantation on these infants is still ongoing (NCT02673788). The same research group is currently conducting a phase II trial to assess the therapeutic efficacy of MSC therapy in preterm infants with IVH (NCT02890953). Of note, the narrow therapeutic window and utilization of brain injury biomarkers should be considered when designing MSC therapeutic protocols for IVH. More well-designed clinical studies to confirm the safety and efficacy of MSC transplantation in IVH patients are necessary.

### Neonatal Stroke (NS)

NS involves injury to the cerebral tissue, usually caused by a disruption in arterial blood flow or major cerebral vein due to the presence of thrombus or embolism. It is a neonatal brain disease with high mortality and morbidity. The use of MSCs has been attempted by Kim et al. for the attenuation of brain injuries in a middle cerebral artery occlusion-induced newborn rat model (26). They observed a reduction in brain infarct volume, improvement of functional tests and enhanced astrogliosis post-MSC transplantation. Similarly, van Velthoven et al. delivered MSCs intranasally to the stroke-affected newborn rats and discovered that the extent of brain injury was reduced, as shown by the decreased size of the infarct, reduced loss of brain matter as well as improved motor functions (27). In another study, exosomes isolated from MSCs were used to treat lipopolysaccharide-induced neuroinflamed 3-day old rat pups intranasally (28). As a result, the expression of inflammatory genes and pro-inflammatory cytokines were suppressed by the exosomes, therefore decreasing the microglia-mediated brain injuries. These exosomes primarily worked by interfering with the Toll-like receptor 4 signaling of microglial cells, hence preventing degradation of NFκB inhibitor (IκBα), as well as phosphorylation of MAP kinase family of proteins.

### Hypoxic-ischemic Encephalopathy (HIE)

HIE refers to a type of brain injury whereby insufficient blood flow is delivered to the brain tissue, resulting in death or severe disabilities such as epilepsy, intellectual disability and CP (29). The current widely used treatment option for HIE is hypothermia, although it only provides a neuroprotective effect rather than a neurorestorative function. Therefore, researchers are working hard to establish a therapy that could improve the prognosis of this neonatal disease.

The combined therapy of hypothermia and UCB-MSCs significantly reduced the cerebral infarcted area and improved the sensorimotor function of rats with HIE (30). This improved diseased condition is thought to be contributed by the paracrine factors released by MSCs, including IGF-1, bFGF, neural cell adhesion molecules, nerve growth factors, and anti-inflammatory cytokines, which stimulated neurogenesis. Moreover, MSCs enhanced expression of genes that triggered cell proliferation while suppressing genes involved in inflammatory responses. Through the inhibition of apoptosis, induction of nerve fibers to remyelinate and axons to regenerate, the administered MSCs were also able to reduce the loss of brain matter while improving sensorimotor functions (31, 32). The potential neurorestorative function of MSCs is further demonstrated in the study by van Velthoven et al. (27). In the control group, inflammatory and cell death activities in the affected rodent brain tissue stopped after 4 days, indicating the therapeutic window to be up to 4 days after the initial attack. However, intranasal transplantation of 1 × 10^7^ MSCs/kg at day 10 post-hypoxia-ischemia attack in the experimental group was still able to limit the brain tissue damage. This implies that tissue reparative activity is ongoing and responsive to MSCs that migrated to the ischemic zone to induce a microenvironmental change which favors neurogenesis (27, 33). Functionality wise, newborn rodents treated with MSCs also showed long-lasting improvement in cognitive and motor functions, up to 14 weeks post-injury (34). In a pre-clinical study, Ophelders et al. reported that intravenous administration of BMSC-derived extracellular vesicles (EVs) to the fetus improved the brain function using a preterm sheep model of hypoxic-ischemic brain injury (35).

### Periventricular Leukomalacia (PVL)

PVL is characterized by a loss of oligodendrocyte and its progenitor cell. This disease is one of the clinical complications of HIE that lead to CP. Other than HIE, the predisposing factors include birth trauma, and immature brain development. In neonates affected by PVL, their periventricular tissue undergoes necrosis while the surrounding white matter experiences diffused gliosis. These changes in brain tissue will disrupt the cognitive, adaptive, motor, and social functions in the infant. Unlike other brain diseases with some limited therapeutic option, there is no specific treatment for PVL except for expectant and supportive management (36). Thus, researchers have begun exploring the use of MSCs for the treatment of PVL.

In the cystic PVL rat model used by Chen et al., excitotoxic ibotenic acid was injected into the white matter of rat brain leading to loss of myelin with transient formation of cysts, activation of microglia and development of CP-like behavioral defects (37). With this animal model, the research group injected the animals intracerebrally with BMSCs 1 day after PVL induction. Results showed that MSCs migrated to the lesion areas in the brain to increase the proliferation of glial cells, hence resulting in improvement of the myelination process with better motor function outcome. Using a different PVL study model that is established through ligation of the left common carotid artery inducing 4 h of hypoxia, the rats were treated with intraperitoneal UC-MSC injection for 3 consecutive days (38). Similarly, functional outcomes improved with decreased microglia and astrocyte activities. Another study by Morioka et al. also demonstrated the suppression of pro-inflammatory cytokines in the affected brain treated with UC-MSCs to reverse the lipopolysaccharide-induced PVL-like injury in newborn rats (39).

## MSCs for the Treatment of Neonatal Gut Diseases

There are several types of neonatal gut diseases that affect the neonate, with higher prevalence in the preterm infant. Currently, MSC therapy has been used to treat NEC in one clinical study involving a neonatal patient. Conversely, several preclinical studies have examined the safety and efficacy of MSC therapy to ameliorate NEC and gastroschisis.

### Necrotizing Enterocolitis (NEC)

NEC is the most common devastating gastrointestinal disease affecting the neonate, with a mortality rate between 15 and 30 % (40). It may affect both full-term and preterm neonates with the latter group having an increased risk. Predisposing factors include prematurity, low birth weight, formula feeding, intrauterine growth retardation, postnatal asphyxia, sepsis, and congenital heart diseases (41, 42). In NEC, bowel inflammation causes necrosis and perforation which may lead to intestinal surgical removal, death or a lifetime severe neurodevelopmental impairment (43, 44)

MSCs have emerged as a promising therapeutic choice for intestinal anomalies such as ulcerative colitis and Crohn's disease (45, 46). However, the therapeutic benefit of MSCs for NEC has yet to be fully explored in clinical setting. A case report of a full-term, 22-day old male neonate weighed 3.350 kg who experienced NEC as a consequence to repeated supraventricular tachycardia (SVT) leading to the resection of 60 cm of the intestine because of gangrene revealed a noticeable improvement after intravenous infusion of MSCs (13). A single dose of 1 × 10^7^ allogeneic UC-MSCs was transfused 4 days after colectomy with no complications. Improved intestinal blood supply was confirmed 3 days post-transplantation, enteral nutrition was resumed at day 8, and the jejunal stoma was closed 46 days post-transplantation. The authors speculated that MSC therapy helped to salvage the remaining of the resected intestine thus prevent short bowel syndrome. Overall, MSC transplantation may offer substantial practical advantages to NEC patients. Nonetheless, findings of this single case report should be interpreted with caution.

In an animal study conducted by Taymen et al. BMSCs were administered to a rat model of NEC (47). The rat showed significant weight gain and improvement in clinical sickness scores after BMSC transplantation. The number of BMSCs homing to the bowel was significantly higher in the treated group. In fact, the severity of bowel damage was significantly reduced in histopathological evaluation. A similar study was conducted by McCulloh et al. using 4 different sources of stem cells, i.e., BMCSs, amniotic fluid-derived MSCs (AF-MSCs), amniotic fluid-derived neural stem cells and neonatal enteric neural stem cells (48). The results indicated that all sources of stem cells reduced the incidence and severity of experimental NEC equally.

### Gastroschisis

Gastroschisis is a full-thickness paraumbilical defect in the abdominal wall that results in herniation of the fetal midgut with a significant mortality rate of 5–10% (49). Feng et al. evaluated the efficacy of intraamniotic delivery of concentrated AF-MSCs to treat gastroschisis (50). The cells were injected into rat fetuses with surgically-created gastroschisis. A significantly thicker muscular layer was observed in the AF-MSC group compared to the control group. It was concluded that AF-MSCs mitigated bowel damage in experimental gastroschisis after intraamniotic injection. A further study was conducted by the same research group but in a larger animal model, i.e., the rabbit (51), which reported similar results.

Since heterotopic cell fusion has been demonstrated between MSCs and various cells, as such, the similar mechanism of protection was also proposed in congenital gut diseases. Nevertheless, preclinical data do not support this hypothesis (52, 53). It seems that MSC homing and paracrine effects are better accepted as the protective mechanism in congenital gut diseases. MSCs may be attracted to the injured site, differentiate to replace damaged cells or interact with native intestinal stem cells to upregulate the Wnt/β-catenin pathway, which promotes auto-regeneration of the intestinal epithelium (54). MSCs secrete a myriad of paracrine factors that promote gut healing and reconstruction. MSC secretome has been shown to improve the severity in a rat NEC model (47).

## MSCs for the Treatment of Neonatal Eye Diseases

Corneal disease is a challenging condition to manage particularly in the neonatal age group. However, successful preclinical studies in the past 10 years have shed some light on the usage of MSCs to treat neonatal corneal diseases. Liu et al. evaluated the efficacy of MSC transplantation in treating congenital corneal disease *in vivo* (55). The corneal disease was simulated using a Lumican null mice model where the cornea is manifested as thin and cloudy due to the disorganization of stromal extracellular collagen matrix and down-regulated expression of keratocan. Results showed that stromal thickness increased significantly and transparency improved in MSC-treated corneas as early as 8 weeks after treatment compared to the control group. This exciting finding has spurred the interest of other research groups to use the similar strategy to treat neonatal patients with corneal diseases.

Mucopolysaccharidosis (MPS) VII is a disease caused by the mutation of β-glucuronidase. Patients with this disease often suffer from developmental defects including short stature, corneal clouding and delayed development. In 2013, Coulson-Thomas et al. transplanted UC-MSCs into the cornea of MPS VII mice (56). MSC-transplanted corneas demonstrated reduced corneal haze 1–3 months post-transplantation. Corneas treated during the first and second month presented significantly improved corneal integrity in comparison to those treated at the third month, suggesting that prophylactic treatment upon diagnosis could prevent the development of corneal clouding. An overall decrease in glycosaminoglycan content was detected in corneas treated at all time points in comparison to the untreated corneas.

Collagen V is a critical protein in the regulation of corneal collagen fibrillogenesis and function development. It has been reported that collagen V knockout stroma demonstrated severe dysfunctional regulation of fibrillogenesis (57). Call et al. treated this congenital and acquired corneal opacity associated with the loss of collagen V in Col5a1^Δ*st*/Δ*st*^ mice (58). The mice were subjected to keratectomy in which the corneal epithelium and the anterior stroma were removed before received UC-MSC transplantation via intrastromal injection. MSC therapy reduced corneal opacity by re-establishing the collagen fibril arrangement via the production of collagen V.

Retinal neovascularization is an unhealthy development which leads to visual diminution and even blindness. Xu et al. studied the therapeutic effect of BMSCs against retinal neovascularization and found that BMSCs migrated and integrated into the host retina, significantly inhibited retinal neovascular tufts and remodeled the capillary network after injection (59). In addition, BMSCs increased the retinal vascular density, decreased the number of acellular capillaries and inhibited retinal cell death.

The therapeutic effect of MSCs in congenital cornea diseases is believed to be attributed to supplementation or replacement of corneal cells. In lumican null cornea model by Liu et al., the injected UC-MSCs acquired the characteristics of keratocytes in which lumican and keratocan were laid down in the stroma cornea (55). The treated animal regained normal corneal thickness and transparency due to reorganization of the collagen fibrils in the cornea. Likewise, MSC transplanted into MPS VII mice cornea also acquired the characteristics of keratocytes. The transplanted cells supplied functional β-glucuronidase to degrade accumulated glycosaminoglycans thereby assisting in lysosome recycling (60). On the other hand, MSCs also secrete paracrine factors that exert anti-inflammation and anti-fibrosis functions, thus suppressing inflammatory neovascularization (61, 62). Through these mechanisms, MSCs can mitigate the inflammation, fibrosis and neovascularisation of the cornea.

## MSCs for the Treatment of Neonatal Heart Diseases

Research has been conducted to investigate the application of MSCs in 28-day old mice/rats for the treatment of heart diseases. In an *in vitro* experiment, it was found that neonatal thymus-derived MSCs (ntMSCs) secreted higher level of sonic hedgehog (Shh) in comparison to neonatal bone marrow-derived MSCs (nbMSCs) (63). Shh is involved in cell growth, cell specialization and morphogenesis of the corpus during embryonic development. Animal studies have revealed that ntMSC transplantation improved the left ventricular function, increased revascularization, decreased scar size and decreased cardiomyocyte death, 4 weeks after infarction. The protective mechanism in this study could be attributed to heterotopic cell fusion. This mechanism occurs when two cells from different lineages merge into one cell, transmitting information and mediators in the process (64). Fusion has been demonstrated between BMSCs with cardiomyocytes, Purkinje neurons and hepatocytes (65). These results warrant further consideration as therapy for heart diseases resulting in early-onset heart failure, such as the cardiomyopathies.

Kang et al. compared the pro-angiogenic, anti-apoptotic and inflammation-modulatory potential of MSCs derived from patients of cyanotic congenital heart disease (C-CHD) and acyanotic congenital heart disease (A-CHD) (66). The purpose of the study is to locate the best source of MSCs for engineered heart tissue treatment (EHT). *In vitro* results showed that MSCs from C-CHD patients expressed higher levels of VEGF-A and VEGFR2, and secreted more pro-angiogenic and anti-inflammatory cytokines under hypoxic condition in comparison to the cells from A-CHD patients. Interestingly, 4 weeks after right ventricular outflow tract reconstruction, cytokine-immobilized patches seeded with C-CHD MSCs exhibited preserved morphology, prolonged cell survival and enhanced angiogenesis compared to A-CHD MSCs. Considering the “naturally hypoxic preconditioning,” MSCs from C-CHD donors might be more adaptable to the hypoxic environment upon transplantation.

## MSCs for the Treatment of Neonatal Sepsis

In sepsis, the host immune system responsible for the clearing of infection may be dysregulated and becomes no longer protective to the host when it is too exuberant. Such an injurious hyperinflammatory response frequently involves excessive release of cytokines and over-activation of the complement system. Unnecessary stimulation of the coagulation system and pro-thrombogenesis is another characteristic of sepsis, which eventually leads to poorer tissue perfusion, cell death and organ dysfunction (67).

A variety of animal sepsis models have been established depending on the use of different bacterial pathogens such as *P. aeruginosa* and *E.coli*, induction with a toxic agent such as lipopolysaccharide (LPS) as well as disruption to the tissue barrier integrity such as cecal ligation and puncture (CLP) (67–70). Using these animal models, the effects of a variety of MSC variables have been tested, including dosage, timing, and route of MSC administration. Reports have shown that MSC dosage as low as 2.5 × 10^5^ cells was effective in reducing the mortality rate of the septic animals though the effect was better when increasing the cell number to 10^6^ cells per animal. Furthermore, the treatment efficacy may be affected by the timing of MSC administration as some researchers failed to identify any positive effects of MSC when administered 6 h post-CLP induction while other identified positive correlation when used at 24 h pre-CLP induction and up to 24 h post-induction. Similar contradictory findings were seen in the route of administration used whereby intraperitoneal injections of MSCs were able to decrease demise of LPS-induced septic mice in one study while the same method resulted in slightly more deaths among CLP-induced septic rats in another investigation (68, 70).

Notably, most of the studies reported that the higher survival rate of septic animals post-MSC treatment is attributable to improved organ function presumably from the MSC anti-apoptotic and anti-oxidant properties as well as reduction of the inflammation-associated tissue injury (68, 71). This is because the administration of MSCs is capable in limiting immune cell infiltration to target organs such as the liver, intestine, lung, and kidney. MSC administration also balances the enhanced expression of pro-inflammatory cytokines seen during the sepsis with higher production of anti-inflammatory cytokines (72).

In addition, MSC may reduce the bacterial burden causing sepsis and increase the survival rate of septic animals. In the study by Zhu et al., treatment with UC-MSCs enhanced the clearance of *E. coli* in 3-day old septic rats with a decreased influx of neutrophils coupled with the increased phagocytic activity of macrophages (73). The antimicrobial activity of MSCs is also due in part to its ability to synthesize anti-microbial peptides. Krasnodembskaya et al. discovered the secretion of LL-37 anti-microbial peptide by MSCs after exposure to *E. coli, P. aeruginosa*, and *S. aureus* (74). Likewise, Lee et al. reported the increased clearance of *E. coli* through the effects of KGF secreted by MSCs (75). A more recent study demonstrated the potential of pre-treating MSCs with polyinosinic:polycytidylic acid (an agonist for Toll-like receptor 3) to further improve its anti-microbial activity (76).

## List of Registered Clinical Trials

Apart from the various completed clinical studies discussed earlier, there are various clinical trials currently registered in the ClinicalTrials.gov database that have yet to publish results (Table 3). These clinical trials use MSCs to treat BPD, perinatal arterial ischemic stroke (PAIS), infantile spinal muscular atrophy (ISMA), hypoplastic left heart syndrome (HLHS), HIE, and IVH. The treatment of neonatal diseases with MSCs is still at the early stage whereby the majority of these trials are either phase I, II or I/II. Of note, MSC-derived EVs, and paracrine factors are also being evaluated clinically for the treatment of BPD (NCT03857841) and HIE (NCT02854579).

## Future Perspectives

### Large-Scale Production of MSCs

A sustainable strategy of MSC expansion is essential to fulfilling the large cell number needed for clinical applications. To achieve a scalable MSC expansion, multi-layered flask, spinner flask, and bioreactor were introduced and gained more attention as a potential for mass production. The multi-layered flask is a static culture system while the spinner flasks and bioreactors are dynamic expansion system. Each of the aforementioned systems has its unique features, whereby the selection should base on preferences or requirements in different situations.

Multi-layered flask offers a simple and straight forward up-scale process. However, due to its larger size compared to conventional flasks, cell monitoring throughout the culture period is almost impossible. Also, the uniform distribution of cells will be relatively harder to achieve and possibly will lead to culture heterogeneity and suboptimal yield of cells (77).

Three-dimensional culture in bioreactor simulates the environment of cells *in vivo*, therefore providing an optimal condition that possibly enhances cellular activities that are not observed in standard monolayer culture (78). Unlike traditional MSC culture protocol, microcarriers are usually added into the culture to provide sufficient growth areas for cells (79). Compared to the multi-layered flask, bioreactor offers better monitoring of cells throughout the culture period and precise control of the culture environment can be done with the aid of computerized sensors in the bioreactor. For more information about the large-scale expansion of MSCs, the readers may refer to the systematic review by Hassan et al. (80).

### Cell-Free Approaches

**Figure 2:**
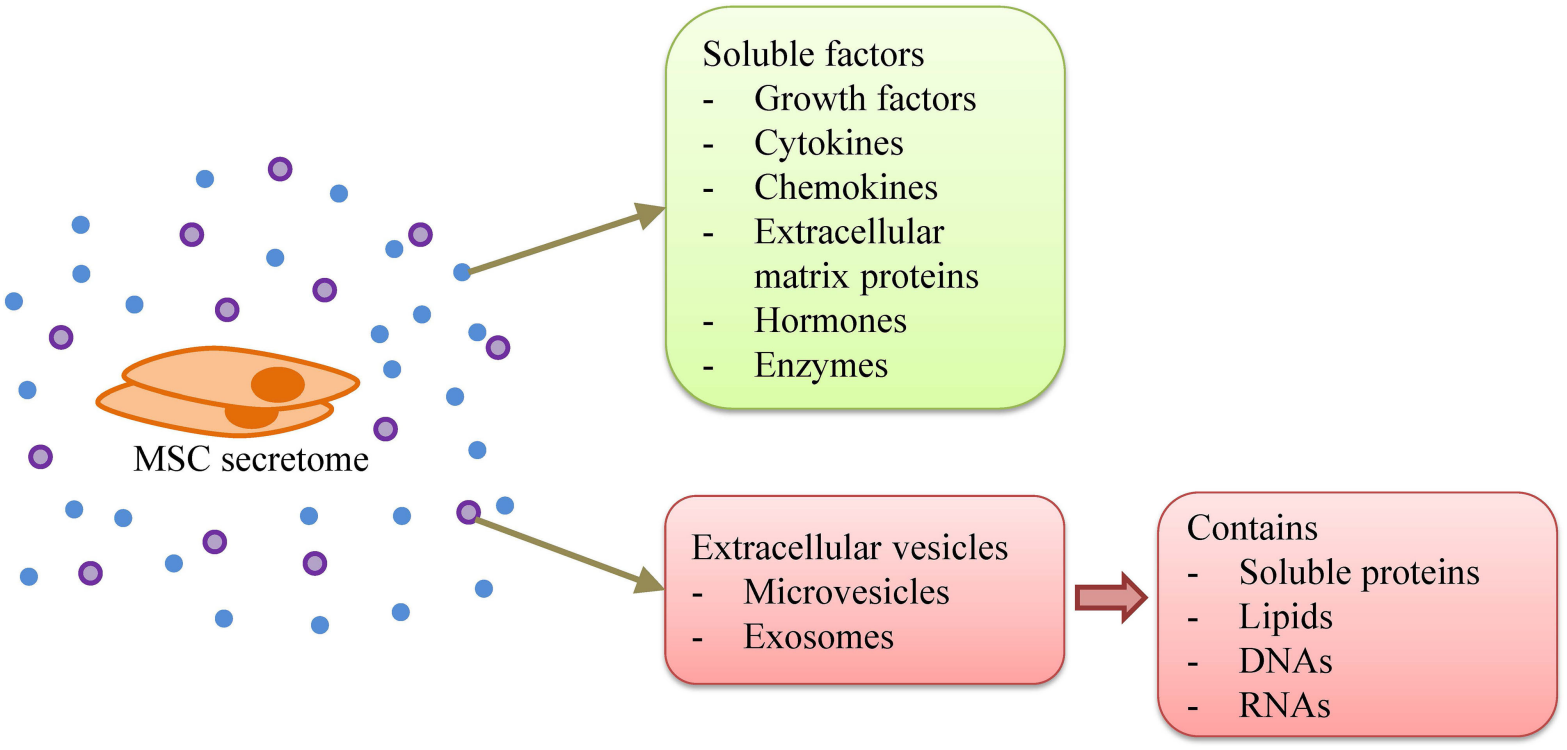
MSC secretome. MSC secretome consists of soluble factors, i.e., growth factors, cytokines, chemokines, extracellular matrix proteins, hormones, and enzymes, as well as extracellular vesicles that contain soluble proteins, lipids, DNAs, and RNAs.

Recently, EVs, including microvesicles and exosomes, have been identified as one of the vital components of stem cell paracrine potency (Figure 2). Many researchers suggested that, if purified and concentrated, the therapeutic potential of EVs can be greater compared to the parent cell (81, 82). In addition, it is still unclear in the long-term regarding the safety of MSC transplantation, e.g., the possibility of malignant transformation. Hence, the use of EVs may be a safer approach and a better alternative to MSC therapy.

EVs are nano-sized particles secreted by cells and are delimited by a phospholipid bilayer. However, EVs do not possess a functional nucleus, hence do not replicate (83). EVs carry various proteins, lipids, and nucleic acids, particularly in the form of RNA, which function in intercellular signaling (84).

EV therapy has been demonstrated to exhibit promising results in ameliorating neonatal diseases in animal model studies (28, 35). A clinical trial (NCT03857841) has recently been developed to evaluate EV therapeutic potential. Nonetheless, there are several challenges to overcome in the bench to bedside translation of EV therapy. One of the major challenges is to identify and quantify the content of EVs, especially the nature of their therapeutically active components. Much effort is made to understand the bioactive elements of these EV-cargoes. EVs are reported to contain proteins, lipids, and nucleic acids, yet there is still a lack of understanding regarding the role of some of these components, particularly the non-coding RNA species. Similarly, the function and mechanism of action of the DNA found in EVs remain unclear.

Besides these cargoes, another vital aspect to consider in EV therapy is the potency. Currently, there are several methods to quantify EV doses based on cell equivalent calculation, protein concentration, EV number and particle size using specialized quantitative analytical measurements, such as nanoparticle tracking analysis (85). EVs are known for having short half-life, as such the therapeutic effect could be short-lived (86). Therefore, a repeat or multiple dosage model is preferred compared to single administration for sustained therapeutic effects over time (87). Sjöqvist et al. observed that repeated administration of EVs was more important than the dose in promoting wound healing in a pig model of esophageal wound repair (88). Furthermore, repeat administration of a lower dose reduces the risk of toxicity and immunogenicity (89, 90).

Another major challenge is to isolate highly purified EVs (91). Currently, purity of EVs is normally expressed as the ratio between protein content and the number of EVs. It has been reported that the purity of EVs is directly related to the isolation technique (91). The isolation technique may impact on EV yield, size distribution and potential biological effects. Currently, many techniques, including ultracentrifugation, size-based filtration, immunoaffinity capture, precipitation and microfluidics-based isolation are used to isolate EVs (92). Different isolation methods yielded a different amount of EVs and Antounians et al. recently reported that significant biological variations were also observed when EVs isolated with different methods were used to treat an *in vitro* lung epithelial injury model (93). The dose-dependent effects have been reported by in several studies (94–96). To date, it is uncertain which isolation technique can provide an optimal yield and the best outcome.

Apart from purity considerations, quality control of EV preparation is also vital. It is proposed that the doses for EVs should be specifically quantified, e.g., by the copies of their microRNAs, so as to reduce the inherent biological variation.

## Conclusion

MSC therapy is a promising treatment for neonatal diseases. The safety and efficacy of MSC therapy have been reported in numerous preclinical and several clinical studies. MSC transplantation can be conducted safely as early as the first month of life. Future studies should be well-designed, i.e., controlled, randomized, involving a larger group of patients, and with a longer follow-up period, to further assure the safety and efficacy of this novel therapy. Besides, more focus is needed to improve on the dosage regimen, to investigate the influence of adjunct therapies on MSC effectiveness and to identify molecular biomarkers to indicate MSC efficacy. In addition, MSC-EV has emerged as a highly potential therapy for neonatal diseases as MSC exerts its therapeutic potential via the paracrine mechanism.

## Author Contributions

FC and JL revised it critically for important intellectual content. All authors substantially contributed to the conception and design of the article and interpreting the relevant literature, drafted the article, and approved the final draft.

## Conflict of Interest

The authors declare that the research was conducted in the absence of any commercial or financial relationships that could be construed as a potential conflict of interest.
